# miR-155: A Novel Target in Allergic Asthma

**DOI:** 10.3390/ijms17101773

**Published:** 2016-10-24

**Authors:** Hong Zhou, Junyao Li, Peng Gao, Qi Wang, Jie Zhang

**Affiliations:** Department of Respiratory Medicine, The Second Affiliated Hospital of Jilin University, Changchun 130041, China; zhouhong0870@mails.jlu.edu.cn (H.Z.); lijunyao191@126.com (J.L.); pengg@126.com (P.G.); wangqi5608319@163.com (Q.W.)

**Keywords:** miR-155, allergic disease, asthma, immune response, glucocorticoid

## Abstract

MicroRNAs (miRNAs), a class of small non-coding RNAs of 18–24 nucleotides in length, function to posttranscriptionally regulate protein expression. miR-155 was one of the first identified and, to date, the most studied miRNA, and has been linked to various cellular processes such as modulation of immune responses and oncogenesis. Previous studies have identified miR-155 as a crucial positive regulator of Th1 immune response in autoimmune diseases, but as a suppressor of Th2 immunity in allergic disorders. However, recent studies have found new evidence that miR-155 plays an indispensible role in allergic asthma. This review summarizes the recent findings with respect to miR-155 in immune responses and the underlying mechanisms responsible for miR-155-related allergic diseases, as well as the similarities between miR-155 and glucocorticoids in immunity.

## 1. Introduction

MicroRNAs (miRNAs) are a class of naturally-occurring small non-coding RNAs 18–24 nucleotides in length [1] and function to post-transcriptionally inhibit expression of their target mRNAs in cells by binding to the 3′-untranslational region of mRNAs, thereby suppressing translation or promoting mRNA degradation [2]. In cells, the precursors of miRNA, pre-miRNAs, can be transcribed from both introns and intergenic regions of the human genome and then form stem-loop structures in the nuclei and are subsequently translocated into the cytoplasm for processing into a RNA duplex [3]. The functional single strand RNA is further dissociated from the non-functional one and assembled into RNA-induced silencing complex (RISC), which is comprised of various proteins, including Argonaute and Dicer, to target and suppress mRNAs with the RISC [4]. miRNA nucleotide sequences do not always have to precisely complementarily correspond to sequences of their target mRNAs, and one miRNA molecule has an ability to bind to various targeting mRNAs [5], indicating the diversity of gene expression regulation. Thus, miRNAs have attracted much research interest in recent decades with regard to their relationship with human diseases [6,7,8,9].

The involvement of miRNAs in human cancer first became evident from a study of chromosomal 13q14 deletion in human chronic lymphocytic leukemia (CLL), which revealed that miR-15a and miR-16-1 were downregulated in 50%–60% of human CLL cases [10]. Further studies confirmed that miRNA played an important role in embryogenesis, organogenesis, cell growth, and programmed cell death, thereby contributing to human diseases, including cancer development [6,11,12]. Cancer researchers thereafter profiled miRNA expression in an effort to classify human cancers and predict survival of cancer patients [13,14]. miR-155, as a functional non-protein-coding RNA located in the BIC gene, was first found accumulated in Burkitt’s lymphoma and in human B cell lymphoma patients [15,16]. Overexpression of miR-155 promotes pre-leukemic pre-B cell proliferation in the spleen and bone marrow, which results in B cell malignancy [17]. In recent years, the expression abnormalities and the key functions of miR-155 were increasingly elucidated in various physiological and pathological processes in the human body. miR-155 expression has been associated with inflammation, immune homeostasis, and cancer development [18,19]. This review discusses the up-to-date findings on the role of miR-155 in allergic asthma and the underlying molecular mechanisms responsible in the development and progression of asthma, as well as the clinical significance and potential strategies in targeting miR-155.

## 2. miR-155 Expression and Functions in Cells

Early Northern blot experiments showed that miR-155 is abundantly expressed in the human spleen and thymus, as well as the liver, lung, and kidney [20]. Thus, miR-155 was thought to play a role in the functioning of the human immune system. Indeed, as an important immune modulator, miR-155 expression is greatly upregulated in a variety of activated immune cells, and miR-155 is mainly involved in immune cell development and the immune response (Table 1). During antigen processing following infection, lack of miR-155 in dendritic cells (DCs) results in failure to present antigens to T cells [21]. During T cell activation, miR-155-deficient CD4+ T cells show increased Th2 subsets in response to IL-4, whereas miR-155 overexpression promotes Th1 responses upon IFN-γ stimulation [22]. It is widely recognized that a Th1 response often plays an important role in cell-mediated immune response in organ-specific autoimmune diseases; however, the Th2 pathway is associated with hormone-mediated immunity and allergy [23]. During the effector phase of the immune response, miR-155 has been shown to be a key regulator for pathogen scavenging and clearance of apoptotic cells. The effector role of miR-155 at this stage is mainly achieved through three aspects: (i) miR-155 is required for antigen specific antibody production and germinal center (GC) response of B cells, essential for B cell maturation, differentiation, and antibody class switching during infection responses [24]; (ii) miR-155 contributes to both anti-microbial and anti-tumor functions in cytotoxic cells such as CD8+ T and NK cells [25,26]; and (iii) macrophages, the most important scavenger cells, express abundant levels of miR-155 after lipopolysaccharide (LPS) stimulation [27]. miR-155 accumulation in macrophages induces a pro-inflammatory M1 response, but inhibits M2 anti-inflammatory activity [28]. Overall, miR-155 seems to be an indispensible transcript in the normal immune reaction and host defense.

## 3. miR-155 Targeting Genes and Related Diseases

**Figure 1:**
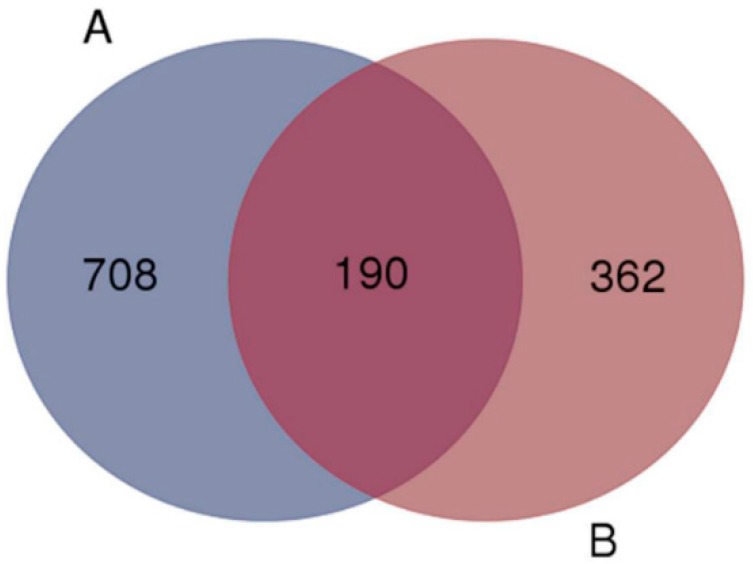
Potential targets of miR-155 identified by miRTarbase and TargetScan. (**A**) miRTarbase identification; (**B**) TargetScan identification. 190 mRNAs were overlapped by bioinformatic analyses, which future studies may wish to explore.

To investigate the miR-155 functional targets, bioinformatic analysis using TargetScan (www.targetscan.org) predicted 552 human mRNAs to be the potential targets of miR-155. Another database, miRTarBase (available online: http://mirtarbase.mbc.nctu.edu.tw/index.php), experimentally validated that 898 transcripts were directly or indirectly modulated by miR-155. As the genes from miRTarBase had a greater chance of being affected by miR-155 targets but not directly by miR-155, another classical target prediction tool TargetScan was added to refine the data. The Venn diagram identified 190 overlapping mRNAs that could be targeted by miR-155 by both methods (Figure 1 and Table S1). These mRNAs encode key regulatory proteins implicated in a number of human pathologies and physiological processes. For example, SPI1/PU.1 contributes to erythropoiesis and hematopoiesis [35,36], and CEBPB suppresses leukemia pathogenesis through the inhibition of proliferation [37]. Both SOCS1 and INPP5D/SHIP1 negatively regulate a series of inflammatory pathway [38,39]. Indeed, FOXO3 and TP53INP1 are known tumor suppressor genes [40,41]. Previous studies have shown that miR-155 levels are elevated in a variety of human malignancies and immune disorders, such as breast cancer [11], systemic lupus erythematous (SLE) [42], and inflammatory bowel diseases [43,44].

## 4. The Role of miR-155 in Asthma

As an important immune regulator, previous studies of miR-155 have focused on its roles in promoting inflammation and Th1 immunity. However, the effects of miR-155 on Th2-related immune response have not been extensively reported, as miR-155 was regarded as a “Th2 suppressor” in initial studies [21,22,29]. The investigation of miR-155 function in Th2 and Th2-related diseases had not been widely reported until upregulation of miR-155 was linked to the development of allergic asthma [45].

Asthma, a chronic inflammatory airway disease, is characterized by reversible airflow limitation, bronchial hyper-responsiveness, or both and has a variety of clinical symptoms—most classically, an audible wheeze. Due to broad and nonspecific definitions, various phenotypes of asthma have been recognized to help classify the disease [46]. To date, asthma can be divided into two different categories clinically according to the dominating immune pathways: Th2-high asthma and non-Th2 asthma. The former exhibits classical Th2 characteristics, such as the accumulation of Th2 cytokines, high levels of eosinophils and mast cells, atopy, and thicker subepithelial basement membrane (SBM). Most importantly, patients respond to corticosteroid therapy [47]. Non-Th2 asthma, however, represents a category of lower airway hyperreactivity asthma in the absence of typical Th2 pathway inflammation. Compared with the Th2 subgroup, non-Th2 asthma is more inclined to show resistance to traditional corticosteroid treatment [47].

As previously shown, miR-155 is involved in the T cell immune response and related cell functions. For example, miR-155 overexpression in CD4+ T cells isolated from splenocytes increases IFN-γ-induced Th1 cell differentiation, whereas suppression of miR-155 with antagomirs promotes an IL-4-induced Th2 phenotype [22,29]. Previously, Rodriguez et al. tested the function of DCs and CD4+ T cells in miR-155 knockout (KO) mice. Interestingly, when co-cultured with miR-155 KO bone marrow-derived mature DCs, ovalbumin T cell receptor transgenic (OT-II) cells exhibited significant impaired proliferation and IL-2 production. This result suggests that miR-155-deficient DCs failed to activate T cells effectively. Furthermore, in vitro culture of miR-155 KO CD4+ T cells with IL-4 stimulation resulted in enhanced Th2 differentiation, accompanied by elevated levels of Th2 cytokines (such as IL-4, IL-5, and IL-10), indicating that miR-155 may negatively regulate Th2 immune responses and inhibit the occurrence of inflammation in allergic diseases [21].

However, more recently, studies of allergic diseases have shown that miR-155 plays an indispensible role in the promotion, rather than suppression, of Th2 pathways. Malmhäll et al. reported that miR-155 KO mice exhibit decreased levels of eosinophils and mucus hypersecretion in allergen-challenged lungs and that Th2 cells and cytokines (IL-4, IL-5, and IL-13) were also reduced, suggesting that miR-155 deficiency is responsible for impaired Th2 activation [45]. This result attracted interest as it was contrary to a previous study by Rodriguez et al. [21] showing that a lack of miR-155 expression promoted Th2 polarization and response. Malmhäll et al. [45] explained that this discrepancy might be due to a different cell sources used between studies as they isolated CD4+ T cells from miR-155 KO mice, which polarized into Th2 cells in vitro, whereas Th2 samples from Malmhäll et al. [45] were collected from allergen-challenged mice. miR-155-deficient mice tend to present with systemic immune malfunctions, including impaired antigen presentation in dendritic cells, which may influence downstream Th2 responses. On the other hand, in vitro polarized Th2 cells seem to be affected by stimulators such as IL-4. This discrepancy was thereafter partially confirmed by Okoye et al., who compared in vitro-generated Th2 cells with those isolated from allergen-challenged mice and found that only 20% of transcripts overlapped between these two sources of cells [48]. These different findings indicate the distinct roles of miR-155 in Th2 development. A lack of miR-155 expression promotes IL-4 stimulated Th2 cell differentiation but prevents the activation of DC-triggered Th2 pathways in vivo.

To support the conclusion of miR-155 accumulation as an indispensible part of Th2-related disorders, further studies in allergic asthma confirmed that miR-155 expression was dramatically upregulated in the lungs of ovalbumin (OVA)-challenged mice compared with non-challenged mice [45]. OVA-treated miR-155 KO mice showed an obvious attenuation of bronchial hyperresponsiveness (BHR), which is one of the dominant features of asthma [49]. Both mucus production and inflammatory cells, in particular, eosinophils induced by OVA asthma, were significantly decreased in the lungs of miR-155 KO mice compared with wild-type mice [45]. This alteration may be due to the damage induced by the Th2 response, as reduced Th2-dependent eosinophils in allergen-challenged miR-155 KO mice may be restored by the adoptive transfer of OVA-specific CD4+ Th2 cells from OVA-sensitized WT mice [45]. Most recently, a novel mechanism was proposed that miR-155 acts as a key positive regulator in allergen-induced inflammation via type 2 innate lymphoid cell (ILC2s, previously known as natural helper cells) and IL-33 [50]. ILC2s are a type of Th2 cytokine producing cells in airway mucosa [51]. The activation of ILC2s contributes to allergic lung inflammation [51]. miR-155 KO mice exhibited deficient IL-33-mediated allergic inflammatory signaling and ILC2s expansion [50]. Alongside asthma, the effect of miR-155 on allergy has also been identified in atopic dermatitis. Compared with healthy subjects, miR-155 was overexpressed in skin lesions in patients with atopic dermatitis, detected in multiple immune cells including T cells, DCs, fibroblasts, and mast cells [52]. In conclusion, increasing evidence shows that miR-155 is a key mediator of Th2-associated allergic diseases.

## 5. Potential Association of miR-155 and Glucocorticoid

**Figure 2:**
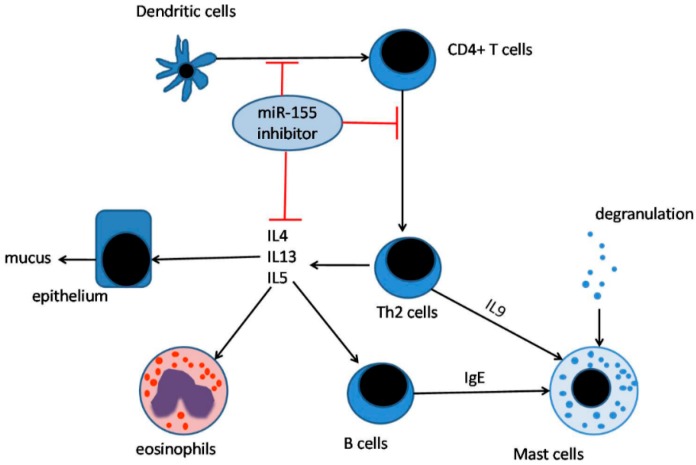
Anti-miR-155 activity in inflammation. miR-155 is able to regulate Th2 inflammation via three major pathways: (i) Prevention of antigen presentation by dendritic cells (DCs); (ii) inhibition of Th2 cell differentiation; and (iii) downregulation of Th2 cell secretion of IL-4, IL-5, and IL-13. Thus, anti-miR-155 could be a novel therapy for Th2 immune response-induced human diseases, such as asthma.

A previous study of the role of miR-155 in Th1/Th2 immune balance showed that miR-155 deficiency in CD4+ T cells selectively biased cells towards Th2 differentiation, whereas miR-155 overexpression favored a Th1 response [22]. Thus, for a long period of time, miR-155 was considered to play a key role in Th1 pathways and interfere with Th2-related activities. However, recent in vivo studies revealed that miR-155 KO in allergen-challenged mice suppressed Th2 lymphocyte-induced inflammation rather than promoting Th2 activity [48,49] (Figure 2). This finding suggests that miR-155 inhibition may be a novel therapeutic strategy for Th2 immune response-induced human diseases, such as asthma.

Interestingly, miR-155 KO affects the innate immune response in a similar fashion to that of glucocorticoids, the latter of which can efficiently suppress Th1-related organ-specific autoimmune disorders and Th2 allergic diseases [45,53,54]. Although a number of studies demonstrated that glucocorticoids act as agonists for the Th2 cytokine response [53,55,56], they are still used as an essential agent for the treatment of allergic diseases. Clearly, this seems paradoxical, as allergy is a Th2-mediated event. Glucocorticoids promote naive T cells into a Th2 phenotype, but the inhibitory effects of glucocorticoids on the production and secretion of Th2 inflammatory cytokines and downstream signaling are independent from T cell differentiation [55,57]. This speculation may explain the role of miR-155 KO in allergic asthma through a steroid-like immune suppression. Further research has shown that CTLA-4, an important inhibitory molecule that functions to regulate allergic response and inflammation, may be involved in T cell-triggered immune responses [58,59]. Indeed, overexpression of miR-155 has been shown to promote proliferation and activation of CD4+ T cells through the targeting CTLA-4 [52]. However, CTLA-4 expression is significantly induced by glucocorticoid treatment, attenuating T cell activation [60].

Despite their Th-related immune regulation, glucocorticoids play a key role in the repression of most immune cells, including macrophages, B cells, DCs, NK, and cytotoxic T cells [61], which is strikingly similar to the effects of miR-155 KO (Table 1). Although the mechanism has not been well discussed in the immune system, this does not keep us from speculating about their potential relevance. A novel theory that glucocorticoids may affect the inflammatory response by suppressing miR-155 has been proposed. As reported, glucocorticoids attenuate LPS-induced inflammation and sepsis via downregulation of miR-155 expression; indeed, the anti-inflammatory role of glucocorticoids can be reversed by forced miR-155 expression [62,63]. Thus, steroid suppression of miR-155 may be a novel functional pathway in immune reactions.

Aberrant miR-155 expression has been observed in a series of autoimmune diseases in the past decade and has been recognized as a potential therapeutic target [64]. However, the study of miR-155 function in anaphylaxis and allergy-inducing cells is still in its initial stages. The mechanisms underpinning the miR-155 pathway are complicated and poorly understood. For example, although mice lacking miR-155 expression show an increase in passive cutaneous anaphylaxis (PCA) [65], this is in accordance with glucocorticoid theory because glucocorticoid was also reported to augment PCA in certain circumstances [66]. Recent studies, however, identified that IgE-mediated mast cell function is impaired in miR-155 KO mice, suggesting that miR-155 is crucial for IL-10-STAT3-induced allergy [67]. Eosinophils, which also play a role in allergy, are also downregulated in miR-155-deficient allergic mice, and the eosinophil chemotactic factor exhibited obvious reductions at the same time [45].

These findings indicate that miR-155 may be a target of glucocorticoids, acting as immune regulators. Thus, inhibition of miR-155 may be further evaluated as a potential therapeutic strategy for the treatment of allergic asthma patients to replace glucocorticoids, although further studies of miR-155 function in allergic inflammation and targets are needed.

## 6. miR-155 Inhibitors as a Novel Therapeutic Strategy

Aberrant miR-155 expression significantly contributes to the development of allergic diseases; thus, modulation of miR155 expression could be considered as an emerging therapeutic strategy for human diseases. However, to date, although miR-155 has been proven to be a crucial regulator in Th2-related asthma, most of the current evidence is obtained from the asthma model of genetically miR-155 KO mice, which cannot be used in clinical treatment. Thus, there is still lack of efficient methods to silence miR-155 expression in vivo. The key challenge is the manner in which to deliver anti-miRNA agents into cells, in addition to the maintenance of their in vivo stability, specificity, and affinity to target mRNA [68]. To solve these pharmacological issues, there are a number of strategies being examined, such as the utilization of antagomirs, locked nucleic acids (LNA), and peptide nucleic acids (PNA), all of which have their own advantages and disadvantages.

Antagomirs are a class of chemically engineered cholesterol-conjugated oligonucleotides [69]. Functionally, antagomirs are anti-miRNA sequences, and it is unclear whether antagomirization with targeting miRNA molecules inhibits miRNA activity or whether they bind to targeted mRNAs, although it is believed that antagomirs may irreversibly bind to miRNA to prevent or inhibit miRNA binding to mRNA [70]. In the past decade, antagomirs were one of the most widely studied anti-miRNA molecules for in vivo purposes. For example, antagomir-122 was applied to reduce serum levels of cholesterol [69]; antagomir-17-5p was used to suppress progression of neuroblastomas [71]; antagomir-10b was shown to prevent and block mammary tumor metastasis [72]. With respect to anti-miR-155, a recent study of a mouse stromal keratitis model of HSV-1 infection showed that subconjunctival injection of miR-155 antagomirs significantly inhibited inflammation and consequentially reduced stromal keratitis lesions and angiogenesis [73]. miR-155 antagomirs have also been utilized to suppress lupus-associated lung inflammation and pulmonary hemorrhages [74].

PNA and LNA are both chemically modified anti-miRNA oligonucleotides [75]. Nanoparticle-encapsulated PNA can efficiently inhibit miR-155 activity in vivo at low concentrations compared with antagomirs, and have exhibited therapeutic activity in the treatment of pre-B cell lymphoma [76]. Similarly, LNA-mediated miR-155 silencing has been revealed to improve the survival of mice suffering strokes [77] as well as being efficacious in the treatment of Waldenstrom macroglobulinemia [78]. The advantage of PNA is that the backbone of PNA contains no charged phosphate groups, leading to a strong binding between PNA-DNA hybrid compared with the binding of normal double-stranded DNA [79]. Similarly, LNA can increase the thermal stability of duplexes after binding to miRNA, LNA is resistant to exo- and endonucleases, resulting in high in vivo stability [80]. Thus, in vivo application of anti-miRNA-155 oligonucleotides could be a potential therapeutic in miR-155-mediated disorders, such as allergy, autoimmune diseases, and inflammation-induced injuries.

However, such approaches are not without potential issues to address; for instance, anti-miR-155 may have serious side effects since one miRNA can generally target multiple mRNAs, indicating that one miRNA silencer may target, or prevent, multiple protein expression. A recent study showed that the efficiency of miR-155 antagomir exhibited variation between different cell types. Intranasal delivery of miR-155 antagomir has been shown to result in good bioavailability in myeloid and dendritic cells; however, the antagomir had limited effects in lymphocytes [81]. As a consequence, it failed to stop Th2-related allergic asthma. Thus, further studies are needed to optimize the safety and efficiency of anti-miR-155 therapy in animal models.

## 7. Conclusions and Future Research Direction

In the past decade, researchers in the field have acquired a significant amount of knowledge and understanding regarding the biological functions of miR-155. Aberrant miR-155 expression alters innate immune responses and contributes to the development of immune related disease and carcinogenesis. However, very little is known about the function of miR-155 in allergic disorders. The current review summarizes the role of miR-155 in the development of allergic asthma, and discusses the potential associations of miR-155 and glucocorticoids. Overexpression of miR-155 is involved in the development of asthma and activation of allergy-promoting cells. miR-155 regulates Th2 immune responses and gene signaling; thus, inhibition of miR-155 expression and activity could be a potential therapeutic strategy for the treatment of allergic disorders.

However, further study of miR-155 function and its respective targets is also crucial and will provide a better understanding as to the molecular mechanisms underpinning asthma pathogenesis. Future research will further investigate and identify the targeting genes of miR-155 in allergy-promoting cells, such as eosinophils and mast cells, in order to facilitate our understanding of the molecular mechanisms of disease development and progression. This can help researchers relate miR-155 expression to the altered expression of different protein-coding genes in each allergic response. Further studies will also provide new information regarding miR-155 expression and the altered expression of proteins, as well as its relationship with glucocorticoids, which may lead to novel treatment strategies for allergic diseases.

## Figures and Tables

**Table 1:** Function and abnormalities of miR-155 in different types of immune cells.

Cell Type	Normal Function	Dysfunction Caused by Overexpression	Dysfunction Caused by Knockdown
B cell	Maintenance of normal B cell differentiation, function and antibody production [17,24,29]	Promotion of pre-B cell proliferation and development of B cell lymphoma [27]	Induction of defects in B cell response, such as reduced antibody production and affinity maturation, decreased memory response [24,29]
CD4+ T cells	Control Th cells bias and IFN-γ signaling	Promotion of CD4+ cells to Th1 differentiation [22]	Promotion of Th2 differentiation in vitro [21,22,29]
CD8+ T cells	Optimization of the effector responses against pathogens and tumors and development of memory response [25,26]	Enhancement of CD8+ T cell response [25]	Reduction of the effector response to infection and tumors and CD8+ T cell proliferation [25,26]
DC cells	Contribution to maturation, survival and activation of T cells [21,30]	Promotion of DC cell apoptosis and enhancement of IL-12p70 production [31]	Induction of DC maturation defects, suppression of DC cell apoptosis, failure to activate T cells but negatively regulate pro-inflammatory cytokines production [21,31,32]
NK cells	Maintenance of maturation, proliferation and effector response	Expansion of NK cell numbers and arrest at their terminal differentiation, but increase in cytotoxic activity against tumor cells and pathogens [33]	Acceleration of NK cell maturation but reduction of their population and impairment of NK cell expansion in response to infection [34]
Macrophages	Regulation of M1 and M2 polarization	Promotion of inflammation and M1 polarization, but inhibition of M2 polarization [26]	Inhibition of inflammation and M1 polarization, but promotion of M2 polarization [26]

## Supplementary Materials

Supplementary materials can be found at www.mdpi.com/1422-0067/17/10/1773/s1.
