# Supplementary material for: miR-155: A Novel Target in Allergic Asthma
Source: Int J Mol Sci. 2016 Oct 24;17(10):1773. doi: 10.3390/ijms17101773 (PMC5085797; doi:10.3390/ijms17101773)
Supplement: Supplementary file 1 [file ijms-17-01773-s001.pdf]

Supplementary Materials: miR-155: A Novel Target in Allergic Asthma

Hong Zhou, Junyao Li, Peng Gao, Qi Wang and Jie Zhang

Table S1. miR-155 targets identified by TargetScan, miRTarBase and both bioinformatic analyses.

Names	Total	Elements
TargetScan miRTarBase	190	LCORL CHD8 TOMM20 SLC33A1 CHD9 ZNF248 IRF2BP2 DNAJB1 C10orf12 PALLD CARD11 GNAS ZBTB38 RAPH1 ETNK2 MSH6 ARL5B CCDC41 MMP16 RHEB TOMM34 MEF2A RICTOR RAB11FIP2 FAM135A ZBTB18 TMEM33 TCF12 KRAS TM6SF1 DHX40 PICALM MYO10 TCF4 FUBP1 ATP6V1C1 SERTAD2 SH3PXD2A UBQLN2 YWHAZ AGO4 CHAF1A ZNF236 MORC3 MEIS1 WWC1 TAB2 NAA50 PRKAR1A CSNK1G2 PHC2 HBP1 SPRED1 ADAM10 KANSL1 MIDN ZNF644 NFAT5 IL17RB STRN3 MAP3K10 ZSWIM6 DMTF1 ITK PDE3A ZIC3 PELI1 CSNK1A1 ARID2 GSK3B SPIN1 TSPAN14 PTAR1 FOXK1 WEE1 PKN2 TPD52 CARHSP1 MYBL1 WBP1L SAP30L VEZF1 EEF2 FLT1 PHF17 RCOR1 SMAD2 CBFB RORA HIVEP2 CHD7 RAP1B SPI1 PEA15 FGF7 RREB1 CBL MYLK S1PR1 TMEM136 PIK3CA NKX3-1 CTLA4 RAB3B SMAD1 ANKFY1 FOS SKIV2L2 SMARCA4 TP53INP1 TSHZ3 PSMG1 FGF2 SKI CPEB4 JARID2 MSI2 SWSAP1 LRRC40 ETS1 COPS3 IKBKE SOCS1 TRIM32 LRRC59 CDC73 RAB5C CAB39 LNX2 NSA2 CDC37 MBNL3 MAFB INPP5D E2F2 PKIA RAB30 CEP41 DET1 UBTD2 C3orf18 BACH1 RAPGEF2 CREBRF SHANK2 PAXBP1 BAG5 KBTBD2 KIF3A HHIP EHD1 HERC4 PALD1 HNRNPA3 N4BP1 PIK3R1 PTPRJ NOVA1 GPM6B CKAP5 TAPT1 CLDN1 SIRT1 SEPT11 COLGALT1 HMGCS1 TLE4 TERF1 ZNF703 FOXO3 KCTD3 APC INADL BCAT1 WNK1 CEBPB TRPS1 CSF1R KDM3A MYO1D RNF123 TADA2B AAK1 RBAK USP8 RCN2 SMAD5 PDE12 ZNF652 MYB
TargetScan	362	SMNDC1 RPTOR PLCE1 KIF26B TNIK RTKN2 ZPLD1 ARRB2 SALL1 C9orf78 SOS1 G3BP1 EHF NUFIP2 UQCR11 AGFG1 ARVCF MEX3B HS3ST3A1 ZNF385D CA13 CACNA2D1 TIA1 G2E3 TFDP2 TRIM23 DUSP7 NFIA ZFP3 RPS6KA5 ATAD2B GPR137B CLCC1 NR1H3 SEC14L5 SAMD12 CTDSPL2 KCNA1 FBXO22 ANKRD6 SPTSSA GOLGA3 GDF6 SYT10 PODN CNTN4 EXOSC7 SMUG1 USP14 MFAP3 SOCS6 BPIFB2 FBXO11 YWHAE EMP2 BDNF LCOR LSM14A C8orf44-SGK3 MIER3 RFX3 GMCL1 C8orf4 ABHD2 SLC12A6 AICDA IPP TMEM202 TRAF3 CNGB3 COL21A1 FAM204A MBD5 HAUS6 METTL21A KDM2A EEFSEC QKI AHCYL2 SGIP1 IL6R UBN2 DCUN1D3 CBFA2T2 ANTXR2 CASD1 DOCK11 PPTC7 NFYA STON1 GRSF1 DHFR ZFP36L2 FBXO33 RSPO2 SLC35E2 USP9X ACVR2A SCN1A TMPRSS11BNL PROX1 HECW2 ALG6 PDE9A UPP2 KSR1 ENTPD7 MYPN SATB2 RDX CXorf23 GRIP1 VTI1A SLA PLEKHG3 DUSP14 ELAVL2 ZBTB16 SOX10 ZNF518B SHH JHDM1D GABRB2 CACUL1 PANK1 LRP1B MYLK4 XIAP CYP7B1 FAM212B ZC2HC1C EBF3 SEPHS1 PLD5 ELF4 REPS2 EBF1 MGP SMS LCA5 BSDC1 ICOSLG PTPN4 DOK6 NMT2 GRIN2A VPS53 OTUB2 ZBTB39 PCSK9 SLC35E2B KIAA0087 GPD1L RAPGEF4 PPP1R3A KPNA1 FAM104A PIAS1 NPHP3 BRD1 FBXO30 GABRA1 NR2F2 SMURF2 FXR1 C11orf30 ENTPD5 ZNF618 NR3C2 CACNA1C UBR1 PAM UHRF1BP1L BTBD3 SRCAP FOSL2 KDM5B KPNA4 H3F3A STARD8 FAM126A INSIG2 SESN3 TCHP EIF5B SLC35F5 F13A1 GATA3 PTPRD STXBP5L C6orf89 NFASC CACNB4 C5orf64 NGEF FAM105A ETV3 G3BP2 SLC2A12 IAPP KIF6 FTO NFIX ELAVL4 EPC2 SCG2 NFIB BCORL1 SPTY2D1 CD99L2 DNAJB7 CLCN5 FLJ20373 FZD5 FGF9 CREB1 FUT9 TBR1 PLAG1 PRMT10 FAHD2A ZNRF3 ZDHHC20 TCEB1 TENM3 MARK2 KLHL8 PPIL2 PRKD1 NRG3 ACVR1 HCFC2 ACTL7A PDE7A C7orf71 MBTD1 IL6ST DCP2 TRIM44 TMEM200C RELA HIF1A WNK3 ATP6V1G1 FBXL17 RNF111 CAMK4 ATXN1L TBX20 DYNC1I1 EN2 RHOQ PSKH1 XRN1 KIAA1468 SOX1 USP48 LRRC8B LRP12 ARID4A PEG3 SP3 ACVR2B GAB3 SUFU OSBPL2 RP11-195F19.5 BRWD3 TM9SF3 RBMS3 SMTNL1 NAV3 FAM83D ZMYND11 TGFBR2 PURB WDR41 TSSK1B ZMYM2 MAP3K7 C9orf69 RGP1 KCTD7 SPIN3 PDS5A TCF7L2 TBC1D15 FAM168A SETD7 PITPNM3 OLFML3 ULK2 SIM2 S100PBP TBRG1 LZIC CYB561D1 HELZ MPEG1 FOXP1 HOOK1 CD47 ABCB11 SOCS5 RNF149 RSRC2 SMARCAD1 TBCK CNIH1 ST8SIA2 NUDT4 VMA21 ASTN1 SHOX SOX11 ACTA1 SYVN1 ECT2L SEMA5A UST RLIM ELL2 SEC61A2 EPAS1 VTI1B PDSS2 NUP43 ELK3 PARD3B CDC42EP1 CSRNP2 TRMT1L GATAD2B SGK3 BCL10 BNC2 THAP6 XKR4 ERN1 ETV1 CTTNBP2NL TAOK1 MID2 NDFIP1 CD274 VAV3 ZC3H12B SATB1 SMIM13 CSF2RB RPS6KA3 BRWD1 LETMD1 AGO3 CDX1 ISCA2 C15orf61 HECW1 RPS6KB1 SPOCK1 KLF3 BAIAP2L1

Table S1. *Cont.*

Names	Total	Elements
miRTarBase	708	RAB2A FSTL1 ERGIC1 EIF2B5 PTN EDN1 ZNF492 DHCR24 FGL2 AIFM1 TAF5L NUP155 CHRDL1 UBE2G1 RAC1 ADD3 ATP13A1 TBC1D14 ERI1 TRIO MITF MATR3 PACSIN2 SH3BP4 CSNK1A1L RDH13 SLC38A5 AP1G1 CTNNBL1 PNPT1 PAPOLA SYNE2 VCPIP1 RAB3IP MMS22L TTC37 SSH2 TSPAN3 KDELC2 LY6K CDH13 TPP2 CTAGE5 FADS1 ATXN10 SLC39A14 SLC12A4 ZNF500 FAM96B TGM2 LUZP1 MECP2 PNPLA4 Sla NASP GRPEL1 STAG2 FUBP3 CYP2U1 INTS6 CLTA CCR9 ATL2 ZNF83 SMAD3 PDCD4 CIAO1 ALDH3A2 MRPS34 PLK1 DDB2 FITM2 MFF ITGB4 LEPREL2 EXOC3 PRKAR2A RAD23B FOXE1 KIAA1715 ZNF148 PGRMC2 CHRAC1 EXOSC2 RING1 SSU72 LPGAT1 RBPJ WHSC1L1 MYO6 CYFIP1 BUD31 SGPL1 MOSPD2 TMEM66 POLE3 CDKN1B PRKCI ADH5 YEATS2 FAM105B CCDC137 FAM98B RTN3 TICAM2 IPO8 TNFRSF10A CAMTA1 MAP3K14 ACOX1 GOLPH3 GMPPA CD36 TRAM1 CCT2 TWF1 SECISBP2 SUPT5H ERBB2IP THRB LARS SNX6 RAB14 STIM1 LUC7L2 EXOC2 B4GALT1 RAI14 ESRRA CYP1A1 PTMS PRSS21 RGL1 HSPB11 ACOT7 ABI2 CDH2 INTS8 ERMP1 PRAF2 HAL KDELC1 UFL1 DSG2 VPS36 GLG1 RHOA CBR4 L2HGDH SLC1A5 CNOT6 NEU1 QRICH1 PCCB ARPC3 C12orf10 TPBG MASTL XPC HSPA4L CARS GNB4 ALDH1A2 AGTRAP AURKA OVCA2 KCTD5 KIF14 SELE ABCC4 PBRM1 NAA16 C16orf62 RNF2 SETD8 NR3C1 CORO1B KRCC1 ASPH COG2 AURKB OXCT1 OSBPL10 LSM3 PPL NFYC KDM1A PDAP1 EIF4E2 MAPK14 MTHFD2 CUL4B RAB27B PACSIN3 VBP1 ZFP36 MTFMT RAD1 TMTC3 MARVELD1 MTRNR2L5 QPCTL AKAP10 GANAB UQCRFS1 ARL10 HLA-DPA1 PLS1 CNDP2 RAB23 AKR7A2 ZNF384 RIF1 RTFDC1 ZNF254 FAM177A1 CPD UQCRB KLHL28 RRAGA IL8 CARS2 PDPR FAM120A ADAMTS4 TYRP1 DPP7 SCD EPRS ANTXR1 STAT3 Cux1 MPZL1 CECR5 PHGDH APAF1 IL13RA1 EIF2B2 WNT5A INTS10 UBA3 MPP2 THOC7 MKLN1 FDFT1 PCYOX1 IMPAD1 UBE2D3 OLR1 GLIPR2 PPFIBP1 C17orf80 Inpp5d REV1 ZNF561 CTNNB1 GALC UBE2J1 NT5E ZNF468 CCND1 NOTCH2 FLNB Hif1a PSIP1 SLC30A7 GABARAPL1 LTN1 LAT2 KCNN3 SIN3A OSTM1 CDK4 DCAF7 AGTR1 KIAA0368 MCAM CDH6 FLI1 FAM199X CISD2 OBSCN RPL39 SPECC1 PODXL FCAMR UBXN2B MAT2B EIF3F ZKSCAN1 TJP1 CSE1L HSD17B7 STX5 Cebpb TYSND1 BCL6 SMAD4 CRAT SLC7A11 DCUN1D2 VAV2 DOCK4 NSUN5 MYC TXNDC12 PNPLA8 MUS81 RP2 SOCS3 CCNT2 KIAA1841 DAG1 SLC9A3R2 DYNC2H1 LONP2 SPCS1 RQCD1 ARFIP2 KLF9 LEMD3 RPAP1 MAVS SLC35A1 CNOT10 MARC1 PRRC1 FAM91A1 HSDL1 KPNA5 GNA13 WDFY1 ATG3 CCL2 SNTB2 WDR82 TRIP13 ZNF611 ARGLU1 S100A11 CLTC GCLC MAN1A2 PDCD10 ALDH5A1 ZNF260 IL6 TWSG1 PSME4 CDC40 IGJ ELK4 CD68 GAR1 CD109 PLXND1 BCL7C ARMC2 TIMM8A SEL1L TRIM24 CHTOP EEF1A2 MYO1E NCKAP1 XPR1 SNAP29 RHEBP1 ANXA2 STK24 GCSAM DOCK1 INPP5F CREB3L2 SLC25A40 GAPVD1 DIAPH3 GNL3L FAM3C INTS7 BRI3BP C9orf142 COL4A2 MRPL18 MTRNR2L9 MOV10 LRIF1 SLC11A2 STXBP2 NUCKS1 SERGEF IKBIP MYD88 TRMT1 WDR11 MARCKS AXL EZH1 KRT80 CMSS1 NAMPT ZNF493 PPP5C CTNNA1 GHITM UGDH AGRN RPS20 SDCBP LCLAT1 PYGL DDRGK1 CLIC4 PHF6 XPNPEP1 DNMT1 EPB41L2 INA SMARCD2 POLR1B UBL3 NUPL2 EIF3A SEC24B TBCA EGFR VPS4B FMNL3 SP1 TNPO1 DENND1B RAB6A EIF4A1 MXI1 TMX3 RBM42 CLINT1 FADD IL2 SRPK2 ATP6V1H IFIT5 DDX3Y CFL2 RIOK2 PSAT1 CALU BTBD1 JUNB MUT PUS7 CCDC82 ZKSCAN5 XPO1 FIP1L1 ZNF300 TTC8 AGL GNPNAT1 CEP55 FKBP3 ARL6IP5 SRSF1 TROVE2 EEF1E1 TNKS1BP1 ARFIP1 DEGS1 ANAPC16 RRM2 HK2 PHACTR2 MGST2 RPRD1A SACM1L TACSTD2 AIMP1 DNAJC2 EIF3J PPP2R2A VPS18 ASB6 FAR1 TMEM167A ICAM1 THBS1 MTAP NOB1 STRBP RBM22 TXNRD1 TMBIM6 SLC30A1 TTF1 UBE2H ATPAF1 GCFC2 NCAPD2 MRPS27 MARC2 ASNS MTRNR2L3 LOC150786 VAMP3 EIF3C CD81 SUZ12 DBN1 SCAMP1 CDK5RAP3 TCEA1 MPP5 HTRA1 SLC25A19 NOS3 CYP51A1 ANPEP RAB34 POLE4 ZNF160 FAM76A DR1 ZNF273 UBXN1 METAP2 NFATC2IP MSH2 METTL7A GPAM CDK2 SELT OSBPL9 RUNX2 DDX10 Csf1r PHF14 OGFOD1 SOX6 TFPI LUC7L3 MRS2 TDRD6 MBLAC2 DDX17 PLEKHA2 UBA2 RAB6C SRSF2 PCCA ANKRD12 PCDH9 UBQLN1 NEUROG1 KANK2 VCAM1 DCAF10 SSSCA1 TMEM123 TMEM194A IFNGR1 OXNAD1 ENTPD1 JUP Sfpi1 C3orf58 INTS4 GLIPR1 Arntl PDK1 SYPL1 YARS SLC39A10 TNFAIP2 RAD51 IER3IP1 MEST DRAP1 CDK5 Jarid2 LPL CAT ZNF98 SLC7A1 BRPF3 ZNF431 Picalm F5 CDV3 VANGL1 KLHL42 CHAF1B NOLC1 CSRP2 SLC35F2 CAB39L DOK2 TIMM13 AKT1 CDC42BPB CIAPIN1 TRAK1 DMD HNRNPA3P1 KLHL5 PPM1G HSD17B12 NES CHURC1 CUX1 POLR2C YBX3 SSX2IP FNDC3B ZNF28 NFKB1 ARL15 KIAA0430 SLC27A2 MRPL16 HAX1 CNPPD1 CTSA TPRKB RARS IGF2R MTRNR2L7 ZNF714 EIF3E PFDN4 TFCP2 NAA25 KRT6B CNNM3 AKR1C3 CLUAP1 ZNF207 EXOC7 FASTKD1 DEK GMPS UPF2 GPT2 CPT1A KIF22 EIF3CL EIF4G2 MLH1 FEZ2 RSF1 LIN7C PSME3 PSEN1 NMD3 CDKN2A PLEKHA5 MECR WRB MEX3C FMNL2 GLB1 EOGT JUN UAP1 LDOC1 ECI1 RETSAT STRN UBE2D2 PAM16 UGT8 NUP62 GATM MCM8 PLAUR NARS NCAPG CUTA FLNA HEATR2 PEBP1 CYR61 AMIGO2 TMOD3 GEMIN5 UBR4 EIF3G GOLT1B ARL5A EDEM3 TBC1D8B ALDH9A1 INPP5A ITGB5 SMARCE1 ZNF678 ARL8B PAK2 DPY19L1 VHL PDLIM5 ABHD16A PCNT UBE2J2 BET1 CD3EAP ACTR2 DNAJC19 H2AFY
